# Adaboost face detector based on Joint Integral Histogram and Genetic Algorithms for feature extraction process

**DOI:** 10.1186/2193-1801-3-355

**Published:** 2014-07-14

**Authors:** Ameni Yangui Jammoussi, Sameh Fakhfakh Ghribi, Dorra Sellami Masmoudi

**Affiliations:** Department of Electrical Engineering, Sfax University, Sfax Engineering School, POB W, 3038 Sfax, Tunisia

**Keywords:** Adaboost, Genetic algorithm, Joint integral histogram, Face detection

## Abstract

Recently, many classes of objects can be efficiently detected by the way of machine learning techniques. In practice, boosting techniques are among the most widely used machine learning for various reasons. This is mainly due to low false positive rate of the cascade structure offering the possibility to be trained by different classes of object. However, it is especially used for face detection since it is the most popular sub-problem within object detection. The challenges of Adaboost based face detector include the selection of the most relevant features from a large feature set which are considered as weak classifiers. In many scenarios, however, selection of features based on lowering classification errors leads to computation complexity and excess of memory use. In this work, we propose a new method to train an effective detector by discarding redundant weak classifiers while achieving the pre-determined learning objective. To achieve this, on the one hand, we modify AdaBoost training so that the feature selection process is not based any more on the weak learner’s training error. This is by incorporating the Genetic Algorithm (GA) on the training process. On the other hand, we make use of the Joint Integral Histogram in order to extract more powerful features. Experimental performance on human faces show that our proposed method requires smaller number of weak classifiers than the conventional learning algorithm, resulting in higher learning and faster classification rates. So, our method outperforms significantly state-of-the-art cascade methods in terms of detection rate and false positive rate and especially in reducing the number of weak classifiers per stage.

## Introduction

One of the most challenging applications in computer vision is detecting objects efficiency in an image frames. Object detection is a computer vision problem having a longstanding history. Though progress has been accomplished, the best algorithms are far from reaching the ease and speed of human being. Object detection is a fundamental problem that merits particular attention since it is the key to solving a number of computer vision applications. However, object detection, especially face detection, is a non-trivial problem due to many associated difficulties and challenges. Given the difficulty of the task, approaches proposed have been somewhat constrained to produce meaningful results. Some of face detection applications are easily defined such as familiar example in digital cameras. Other applications are far broader in scope, such as human-robot interaction for medical assistance.

As an expanding hot topic, a number of promising face detection techniques has been developed since decades ago. The basic principle is to search everywhere in a video stream or an image and detecting wether or not faces exist and so finding its locations. Early works (before year 2000) had been surveyed by Yang et al. (2002) and classified into four main categories: knowledge-based, feature invariant, template matching and appearance based methods. However, these categories have a large overlap especially for template matching and knowledge based. Another classification proposed by Gong (Hjelmas 2001), divides the face detection approaches into two groups: local feature-based and holistic-based. But, skin color based approaches are not included in this classification. A classification rule is adopted recently (Dengpan 2010), so that face detection methods are classified firstly into two groups: rule-based and learn-based.

### Rule-based methods

In a rule-based method, face detection is achieved based on some knowledge or predefined rules. Facial features, texture, skin color and predefined templates all belong to this category. Knowledge-based methods are the early proposed methods in face detection. They make use of the human consent based on the constitution of face images using rule-based methods. This method is characterized by its easiness to come up with simple coded rules presenting features of a face and works well under uncluttered background. In these approaches, given an input image, facial features are extracted and then faces are identified based on the coded rules. The major faced problem is that the performance depends on the geometrical rules and it is difficult to translate human knowledge into precise rules. If the rules are detailed, they fail to detect faces and general rules can produce many false positives. Moreover, it is also extremely difficult to enumerate all the possible cases by simple rules. At the highest level, the rules are general descriptors of what a face looks like. However, at lower levels, rules rely on details of facial descriptors.

### Learn-based methods

In contrast to template matching, templates in learn-based methods used for face detection here, are learned from training sets to represent the variability of facial appearance, rather than predefined by experts. These methods which consist on extracting features from pixel intensity are based on the machine-learning approaches using a large set of face images in the training process. Besides, they rely on statistical analysis to discover characteristics of face and non-face images. Learn-based is the most adopted approach in recent advances in face detection and has demonstrated good results in terms of robustness and speed. Besides, these methods can be extended to detect faces in different poses and orientations. However, they present some difficulties: the huge number of positive and negative samples that we must provide, the long consuming time according to the training process and the detection of the face in the image needs to search over space and scale, etc. Despite these difficulties, these methods have shown higher performance than the other methods, due to the rapid growing in data storage and computation power resources.

If one were asked to name face detection techniques that have the most impact in this field, it will be most likely the Rowley and Canade face detector based on neuronal network and more recently the seminal work of Viola and Jones based on Adaboost.

The boosting statistical approach is firstly used by Viola and Jones in their face detection framework (Viola and Jones 2001). It can be explained by the process of constructing strong hypothesis through linear combination of weak ones. Since Viola and Jones framework, contributions are concerned either with improving the feature extraction process or searching to improve the boosting alternative. In this work, we restrict ourselves to improving the performance of Adaboost face detector by the amelioration of both the feature extraction process and the training process. The following section is intentionally restricted in scope to provide an overview of Adaboost based face detector.

## Adaboost based face detector

A variety of face detection techniques have been proposed over the past decades. Boosting-based method is considered as the de-facto standard of face detection. The Adaboost technique (Adaptive Boosting), initially proposed by Yoav Freund and Robert Schapire (1995), presents a popular machine learning technique for selecting a set of more performing weak classifiers from a pool of over complete weak classifiers (Freund and Shapire 1995).

For the training stage, a very large set of labeled samples is used to identify the best weak classifiers, and a strong classifier network is constructed by a weighted linear combination of these weak classifiers.

Combining the cascade structure and Haar-like features, Adaboost based face detector performs well the detection task in terms of speed and precision which make it suitable for real world applications (Viola and Jones 2001). Capable of eliminating most of the negative samples efficiently, the merit of boosting-based detector still shines (Curuana and Niculescu-Mizil 2006). Besides, the performance of Adaboost based face detector can be enhanced by the use of more sophisticated features and other more accurate learning methods. According to a literature review, the learn-based methods are more attractive. Nearly, the best face detectors fall within the state-of-the-art of learn-based methods. However, the machine learning methods suffer from the huge number of required training samples which makes it more difficult to build an automatic face detector system without human supervision or user’s intervention. In this work, we restrict ourselves to ameliorating the Adaboost based face detector by searching for efficient features and improving the selection process.

### The Adaboost algorithm

Each hypothesis in the training algorithm is constructed using a single feature. The algorithm is described in the following:

- Given a series of samples (*x*_1_,*y*_1_),........,(*x*_*n*_,*y*_*n*_) where *y*_*i*_=0,1 for a binary outcome.
- Initialize weights  for *y*_*i*_=0,1 respectively, where m and l are the number of negative and positive samples.
- For *t*=1…*T* :
  1. Normalize the weights
    so that *w*_*t*,*i*_ is a probability distribution and *i* is an image index.
  2. For each feature *j*, train a classifier *h*_*j*_ (each classifier corresponds to a single feature). The error is evaluated with respect to *w*_*t*,*i*_, .
  3. Choose the classifier, *h*_*t*_, with the lowest error *ε*_*t*_.
  4. Update the weights:
    where *e*_*i*_=0 if sample *x*_*i*_ is classified correctly, *e*_*i*_=1 otherwise, and
- The final strong classifier is:
1

where *α*_*t*_=*log*(1/*β*_*t*_)

### The cascade structure

In the Viola and Jones framework, the Adaboost learning method is employed using Haar-like features as weak classifiers, in order to obtain a strong classifier stage. Instead of learning a single classification stage, a cascade of strong classifiers is learned. The training of the cascade was achieved using few thousands of positive samples associated with a very large pool of negative samples (Viola and Jones 2001). For each stage, the all available positive samples are used with the negative samples that survived the previous stage (detected as false positives).

**Figure 1:**
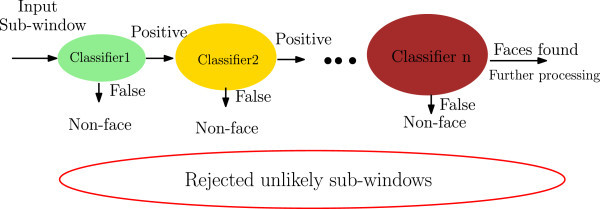
**The attentional cascade structure.**

In the earlier stages, the strong classifier obtained by few number of weak classifiers are capable of rejecting the majority of negative samples. Later stages are concerned with more and more complex classifiers to achieve low false positive rates (cf Figure 1). Going forward in the cascade structure, the task becomes more difficult because only hard samples go through the later stages and each classifier is slightly more complex than the earlier ones. Thus, through the cascade stages, the attention of such system focuses on more reduced and relevant sub-windows. Preserving complex stages to the later stages in the cascade for processing hard samples, reduces significantly the computing time. The major advantage of such structure is then improving the speed of the face detector given that the earlier stages reject the unlikely samples and the last stages focus on the sub-windows most probably containing faces.

## Adaboost based face detector optimization

### Features in Adaboost based face detector

There exists a wide range of features that are chosen according to the specific environment where the detector operates (Zhang and Zhang 2010). Some features are more complex and more expensive to compute than others. Feature complexity is generally dependant to the requirements of the detector in terms of accuracy and speed.

Histograms are among the most prominent features used in many computer vision applications from object based retrieval, to segmentation, to object detection, to object tracking, etc. A histogram can be defined as an array of numbers, in which each element (bin) corresponds to the number of occurrences for a given data value. However, single feature histograms represent generally only global feature composition and images with very different appearances can have the same histograms. On the one hand, joint histograms come to solve this problem by incorporating additional information to histograms.

On the other hand, the computational complexity, once histogram extraction is achieved intending for comparison, is one major bottleneck of histogram based methods.

There are some attempts to deliver accelerated alternative techniques to the basic exhaustive computation. Computer vision problems that look for the optimal solutions, for detection and tracking, still require a theoretical breakthrough in histogram related computing. As a solution, the integral histogram is proposed to allow faster extraction of rectangular region histograms. Accordingly, it is computed by intersection of the integral histogram at the four corner references using simple arithmetic operations (Porikili 2005). In the literature, various efforts have been made to improve the feature computation speed and so the final detector’s speed. In fact, inspired from the integral image representation, other representations are proposed to facilitate the computation of different values under a square region.

In this paper, we try to use joint histograms by the way of new integral data structure for fast feature computation. Taking into consideration the advantage of integral representations, we make here use of both integral image and integral histogram using a new method referred to as Joint Integral Histogram (JIH).

### JIH based feature extraction

The Joint Integral Histogram is inspired by both the integral image and a related image processing task transformation. In a JIH, the value at each bin is accordingly determined by two characteristic images *f* and *g*. For each bin *b*_*i*_, for each pixel (*x*,*y*), we define a joint histogram matrix , given by:
2

In this matrix, the function *g*(*x*,*y*) determines which bin to increase and *f*(*x*,*y*) determines the value to increase at that bin. The equation of JIH is constructed as follows:
3

**Figure 2:**
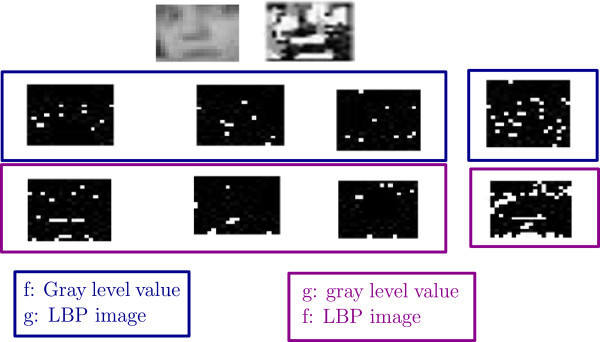
**JIH illustration on a sample face image of two possible combinations: f represents the gray level pixel values and g represents the LBP image values and reciprocally.**

As the equation shows, the JIH is composed of a combination of an integral image and an integral histogram. The contribution of (*x*,*y*) to JIH is jointly determined by two functions *f*(.) and *g*(.). In a JIH, instead of remembering bin occurrences, the value at each bin indicates an integral defined by two signals. Once the JIH is computed, joint histograms over a rectangular region *Ω* can be computed in a constant time:


As in JIH we can make use of two sources of information, inn this work, we further exploit Local Binary Patterns (LBP) 2002 potentiality in extracting orientation and gray level values independent features within the JIH extraction method. The JIH represents a distribution of the different values of bins. Thus, the JIH structure contains more details than initial functions *f* and *g*. Once the JIH is computed, we can extract the mutual information within a sub-region from the whole image. The JIH computation for a face image of two possible combinations is illustrated in Figure 2.

The best features which separate face and non-face samples are chosen by Adaboost learning algorithm. Further confirmation of the extracted features was achieved by a comparison with the conventional Haar-like features.

### Genetic algorithm optimization

In Adaboost face detectors, some limitations which are related to the learning process are faced. In this work, we make headway toward overcoming these limitations by reducing the number of weak classifiers in each stage taking into account that often some of the selected features are irrelevant and do not contribute to the progress of the training process. In fact, we try to investigate the Genetic Algorithms (GAs) in the training process to optimize the detection performances.

Recall that the weak classifier functionality is to find a weak rule *H*_*t*_ :*X*→*Y* which is appropriate for a distribution *D*_*t*_. But what we mean by appropriate? The quality of a weak classifier depends on its error, according to the weights:
4

The error is measured with respect to the distribution *D*_*t*_ on which the weak learner is trained. In practice, the weak learner is an algorithm that use the weights *D*_*t*_ on the training samples. For each iteration in Adaboost algorithm, a new weak classifier is selected according to the error criterion. In some cases, no improvement made related to the detection rate or false positive rate. We note that some weak classifiers greatly enhance the performances but other features do not contribute and even end up with a performance drop.

This is can be explained by the fact that, often some of selected features although leading to lower errors, are irrelevant which increases the training time and memory resources. A key question here is how to add relevant features without degrading the training performances ?

This limitation motivated us to find a search technique of weak classifiers that outperforms the solution based on lowering the classification error. A face detection task is considered as a classifier training problem, searching the parameters for a best modeling of a given training data. In the standard model, we need to specify many parameters and then estimate their values from training data. When these standard models are simple, it is possible to find their optimal parameters by solving equations explicitly. However, when the task becomes more complex, it is very difficult to find the optimal parameters. In our case, selected features by Adaboost within a single stage are dependant on each others and there is no analytic relation between the number of features and corresponding detection performance. Thus, the optimization task is non linear, hard and then seems to be suitable to be treated by GAs.

### Training procedure analysis

**Figure 3:**
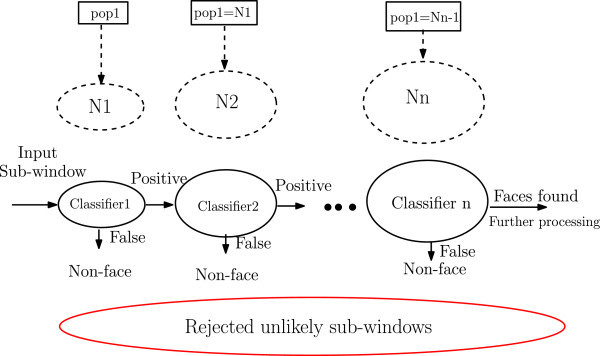
**The training process based optimization.**

Generally, in the cascade training procedure, we have several parameters to optimize such as: the detection rate, the false positive rate, the number of features in each stage, the number of stages in the cascade. According to the literature review, there are some attempts to investigate the GAs in order to ameliorate the face detection algorithm (Chen et al. 2004) (Treptow and Zell 2004) (Yalabik and Yarman-Vural 2007) (Zalhan et al. 2007a) (Zalhan et al. 2007b) (Yang et al. 2010). These parameters requires a multi-objective method to be optimized. Searching for simplicity, *ε*−constraint method was adopted in our work (Coello 1996). The entire *ε*−constraint based training procedure is summarized as follows:

1. Representation: Each individual in the population corresponds to a number of weak classifiers to construct a strong one using Adaboost. The number of weak classifiers by individual is denoted by *T* which is variable from one stage to another. In order to preserve the general cascade behavior, that is the number of features increases going forward in the cascade, we fixed the maximum number of classifiers per stage (denoted by *T*_*max*_) in function of the stage’s index *i* by: *T*_*max*_=*i*∗10.
2. Construction of the initial population: As explained above, the maximal dimension of strong classifiers is initialized in GAs and depends on the index of the current stage. In genetic based Adaboost, initial individuals are of variable length. We denote by *T*_*i*_ the number of genes of individual *I*_*i*_, with *T*_*i*_<*T*_*max*_. Each gene of *I*_*i*_ is denoted by *g*_*i*,*j*_, *j*∈{1,2,…,*T*_*i*_}. For the first stage, the initial population is generated randomly. For the subsequent stages, the generation of the initial population is inspired from the nested cascade behavior (S~ochman and Jir~i 2004), that is the initial population of the current stage is the strong classifier of the previous stage (cf Figure 3).

3. Fitness computing: This function makes it possible to evaluate the effectiveness of the chromosome solutions. It depends on criteria which should be maximized or minimized.

The GA objectives are defined based on the method *ε*−constraint. In general, the selected objective is the one that the decision maker wishes to optimize in priority:
5

In our work, we aim to optimize the detection performances: the false positive rate and the detection rate. An analysis of the different possible alternatives is processed in the following section.

4. Reproduction: For this step the elitist method is used, which is intended to prevent the lost of the best individuals. Thus, technically, best individuals are reinserted in the future population and the remainder of the future population is constructed based on the wheel selection method.
5. Crossover: The crossover is an exchange per blocks of elements between two chains to generate one or two others of them. A site of crossover is randomly selected over the length of each parent chromosome and a cut of the chromosome is done. This cut produces two pieces which can be permuted. The resulting children chains contain each a piece inherited from each parent.
6. Mutation: In binary population, some bits of population are chosen to sudden mutation, according to mutation’s probability. Their values are then reversed.
7. Population sorting: In this step, we perform the union of populations before and after genetic operations (crossover and mutation), then we sort them according to the false positive rate, the best half of the resulted population are chosen to participate in the future generation by the elitism mechanism.

## Experimental results

### Experimental database

The CMU PIE database consists of 41,368 images of 68 people. Each person is under 13 different poses, 43 different illumination conditions, and with 4 different expressions.

**Figure 4:**
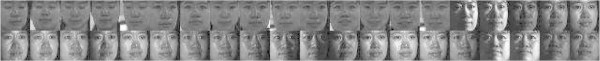
**Several sample face training images from PIE CMU database.**

Faces were cropped and resized to images of size 32×32 pixels. Some sample face images are illustrated by the following figure (Figure 4). The number of non-faces is higher than the number of faces in order to represent the disparity of existing patterns on real images. In fact, in real images there are much more non-face patterns than face patterns. The choice of the number of images in the training database affects the system performances.

### Feature evaluation on a single-node detector

To evaluate the global performance of the proposed feature, the detection rate and the false positive rate of the JIH based features is compared to the conventional Haar-like features using 2500 faces, 2500 non-faces for the training data and 500 faces and 500 non-faces for the test data (Table 1). The obtained results show that the JIH based features are more powerful than the Haar-like features in terms of detection rates and false positive rates.

**Table 1:** **Comparison of the DR and FPR between JIH and conventional Haar-like features**

Number of features	Haar-like		JIH	
	DR	FPR	DR	FPR
10	99.17%	30.4%	99,58%	0%
50	99.17%	1.4%	99.9%	0%

### Training algorithm evaluation

In our work, as mentioned above, we adopted the *ε*−constraint method in our optimization problem. For this method, we have to choose which parameter is considered as the objective function and which one is considered as a constraint. To validate our choice, experiments are conducted to train a single stage with fixed number of features using 5000 faces and 10000 non faces and we compared the obtained performance results (Table 2).

**Table 2:** **Comparison of the performance results between different possible alternatives**

Constraint	***F*** ***P*** ***R*** < 0***.***5		***D*** ***R*** > 0***.***99	
**Fitness**	**max(DR)**		**min(FPR)**	
NB	DR	FPR	DR	FPR
10	99.37%	64.8%	99.37%	41.6%
20	99.17%	20.4%	99.17%	16.8%
50	99.17%	3%	99.17%	1.8%

Given these results, we can conclude that the performance results obtained for the different alternatives are almost the same. With the second alternative, we obtain results slightly better than the first one especially in term of false positive rate. Another parameter that should be taken into consideration is the number of features in each stage. This parameter is very important and contribute well on the speed of the final detector.

**Figure 5:**
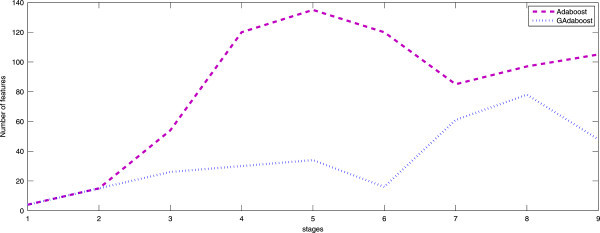
**Comparison of the number of selected features between Adaboost and our proposed training procedure.**

To evaluate the global performance of the investigation of the GA within the Adaboost framework, we process by training a cascade with the conventional Adaboost and a cascade with our proposed training procedure (Figure 5).

Given these results, we can conclude that the investigation of the GAs in the Adaboost process ensures the optimization of the system performances given a number of features. It was achieved by selecting the most relevant features and eliminating redundancy. In what follows, we conduct some experiments to show the influence of some parameters on the adopted training procedure.

#### Influence of the initial population

**Figure 6:**
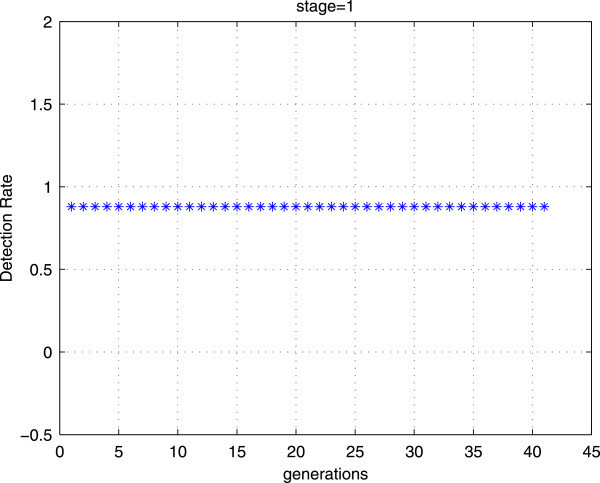
**The goal performances are obtained with a small number of generations (42 generations and the latest value with which the algorithm converge is not represented).**

**Figure 7:**
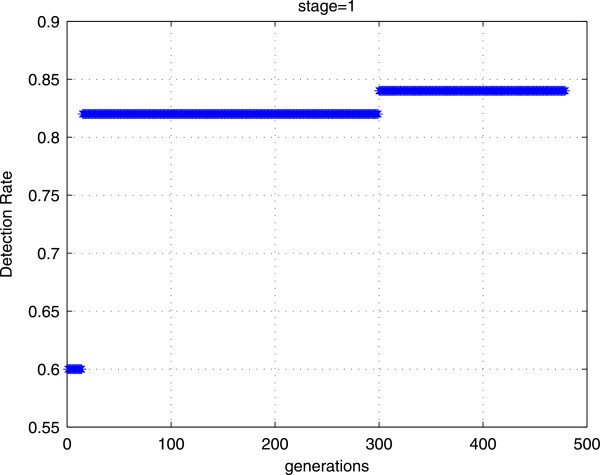
**The goal performances are obtained with big number of generations (more than 450).**

Experiments are conducted to train a single stage with our proposed training method using two different initial populations with the same number of individuals (cf Figures 6 and 7). The obtained results show that with the first initial population, we can achieve rapidly the goal performances. However, with the second initial population, the number of generation is more and more important. Thus, the initial population has a great impact on the convergence of the GAs.

#### Influence of *T*_*dim*_

**Figure 8:**
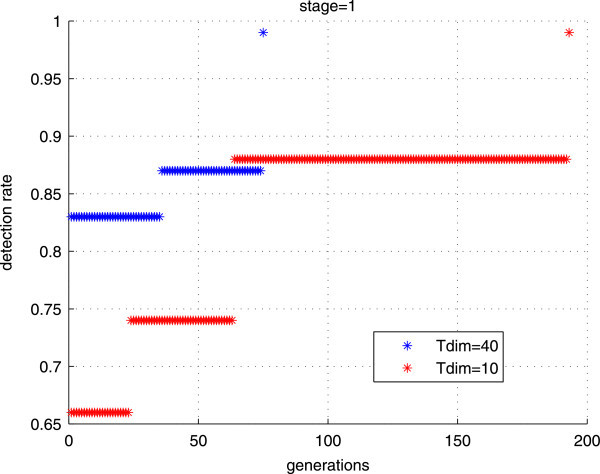
**The obtained detection rate through generations for** ***T***_***dim***_ **= 10 and** ***T***_***dim***_ **= 40.**

As shown in the following figure (Figure 8), we notice that the choice of the individual size *T*_*dim*_ has an impact on the training process. Given two different values of *T*_*dim*_, the performance goals are achieved rapidly for *T*_*dim*_=40 with almost the same number of features.

Given these results, we can note that the choice of *T*_*dim*_ larger than we require in the stage can help GAs to converge more easily and rapidly.

### Training a cascade

**Figure 9:**
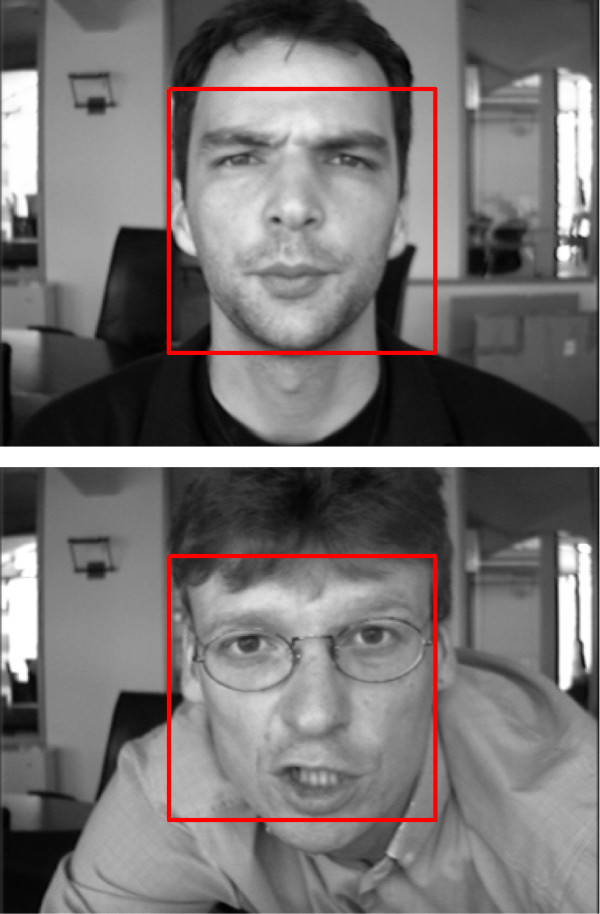
**Example of detection results on BioID database.**

For each stage classifier, the minimal detection rate is 0.99 and the maximal false positive rate is 0.5 on the validation data set. For the learning process, we have to start with a big number of negative samples. Then, at each stage, only the samples that are classified as positive are kept on the subsequent training set. Thus, the next stage in the process is trained to classify the examples that have been misclassified by the previous stages. Furthermore, a few number of hard samples (like faces) are left to the latest stages of the cascade. Starting with 5000 faces and 10000 non faces, we obtain a cascade composed of ten stages and 584 features. Figure 9 show some detection results on BioID database.

## Conclusion

In this paper, we have proposed the investigation of the GAs within the Adaboost training process for efficient feature selection. Instead of selecting sequentially weak classifiers with Adaboost training process, we proposed to select them at the same time to construct a strong classifier for each layer. The maximum number of features for each layer is fixed in advance and then the features are selected without redundancy. In the same context, to further improve the training process, we have proposed a more powerful features using JIH. Our proposed method based on GA and JIH makes possible the reduction of the number of features for each layer and the amelioration of face detector performances. There is still room to further improve the system performances, so our future work consists on applying a multi-objective method.
